# Supplementary material for: Sky and wire: A UAV-BPL synchronization algorithm for information exchange in herding operations
Source: PLoS One. 2025 Oct 22;20(10):e0334888. doi: 10.1371/journal.pone.0334888 (PMC12543189; doi:10.1371/journal.pone.0334888)
Supplement: Attachment — Submitted filename: Comments.pdf [file pone.0334888.s001.pdf]

This paper proposes a synchronization algorithm for dairy farm management that integrates UAVs with BPL to optimize data collection, either by maximizing coverage or by prioritizing urgent data and energy efficiency. I appreciate the authors efforts and the interesting topic addressed. However, the paper lacks valid simulation settings and in-depth discussion and detail in several areas that need to be further explored. There are also a number of weaknesses that require serious and careful revision. Below are some of my observations and comments, which should be thoroughly addressed and incorporated into the manuscript, not just provided as responses.

1. The abstract needs improvement in terms of language, flow, and clarity. Please revisit your abstract and rewrite it professionally and concisely, clearly highlight your objectives, contributions, and methodology.
2. You mentioned two scenarios in the abstract, but only one is described, while the other is unclear and appears mixed into the description.
3. The entire paper needs thorough English proofreading, as there are several unclear statements, improper flow, and ambiguities throughout the text.
4. Apart from satellite communication, the paper requires more statistical analysis regarding the cost-effectiveness and affordability of using UAVs compared to traditional wireless monitoring methods.
5. Please correct "$Ei$" in "Where $Ei(t)$ is the energy level" to "$E$j" on page 8.
6. Please remove the repetition in the fourth paragraph of Section 3.1, starting with "The aim is to minimize the total distance UAVs travel to…"
7. Please revise the section on "The Coverage-Driven Scenario (Scenario 1)," its equations, and the algorithm, as there are some contradictions between your descriptions, equations, and algorithm. For example, there is inconsistency regarding whether UAVs collect data from sensors or relay points.
8. It is not clear how BPLs are used, what standards are followed, and how they operate. Please elaborate and provide technical details on BPL operation and its integration with UAVs.
9. There should be a separate section explaining the "UAV-BPL synchronization" in detail, including how it is achieved and what are the transmission protocols used from sensors to BPLs, through BPLs to its relay points, and from relay points to UAVs.
10. What are relay points made of? Please clarify.
11. There seems to be a contradiction between your objective equation no. 3 and the description of xij and dij . Based on equation 4, I assume "xij(t) is the binary decision variable for UAV movement (xij(t) =1 if UAV j collects data from source i, 0 otherwise)," and "dij(t) is the distance from UAV j to the BPL relay serving source i" correct? This is different from your

description. Please revisit all your equations and descriptions and clarify the relationship between variables for the reader.

12. Also, in Algorithm 1, there are contradictions: “$dij(t)$” is used as the distance to the nearest relay, while xij(t) is used as a decision variable. Please ensure consistency.
13. I did not see a check for CBPL(t)≥Cmin before relaying data, as indicated in line 17 of the algorithm 1.
14. It is unclear what is considered a data source. In Section 3, you mention data sources 1 to N (sensors) creating packets and sending them to relay points, but later in the scenario description, it seems you refer to relay points as data sources visited by UAVs. Please be consistent.
15. I did not see any UAV energy/range check in Algorithm 1. Is it used as input? Why?
16. I suggest using a threshold symbol other than $\emptyset$, which means “empty set”
17. In Algorithm 2, the energy consumption rate is used, but how is it determined? Please explain or provide a proper citation.
18. Please remove the two captions “Table 2 Coverage-Driven” and “Table 3 Energy-Efficient Priority-Driven Exchange.” You can refer to them as Algorithm 1 and 2, which are already captioned.
19. How do you define "critical" data sources, or in what cases we know that it is critical? and how is R chosen?
20. Does your model allow a UAVs to visit more than one data source per time step? If so, should there be a constraint on UAVs capacity or energy?
21. In your simulation, you mention a 200x200 area. Is this in meters? If so, this is a small-scale farm that could be covered by traditional wireless methods, while your solution aims to address large-scale farms. How do you validate that your solution meets your design objectives?
22. This simulation is conducted for a single iteration of target monitoring, which definitely results in higher performance metrics and very low energy consumption. For validity, the simulation should be extended to cover a specified duration that emulates several hours or days of operation.
23. Your simulation lacks context and a valid environment. Please mention your simulation environment (tools, libraries), and explain how you simulate UAVs and BPLs, the standards and protocols assumed for communication and transmission, and the integration of UAVs and BPLs. Even though you have “Communication Technologies Comparison” in you related work section, but these were absent in you simulation part.

24. The simulation also lacks in-depth analysis, comparison, and justification. It appears that results are based on set parameters without reference points. Please provide more analysis and justification.
25. In your simulation results, you claim "100% coverage of all 50 data sources" and "highlighting its energy efficiency". This result is expected given your design, implementation, and parameter selection, but there is no comparison with other solutions to demonstrate novelty. Please provide comparisons with some existing approaches and highlight where your solution is superior.
26. What energy consumption model did you use?
27. How did you choose the parameters in Table 4 (UAV range, energy rate, redundancy factor, critical data, etc.)? Are they based on datasheets, chosen arbitrarily, or taken from references?
28. In Figure 1, the legend does not reflect what is shown in the figure. For example, all UAVs 1 to 5 have the same line style and color, so why you listed them separately? Also, the colors (green, blue-green, yellow, etc.) given on the scale 0.2 to 0.8 are not explained.
29. I also suggest diversifying your results presentation by including tables along with graphs.
30. Please use a standard referencing style according to the journal guidelines.
