# Supplementary material for: Sky and wire: A UAV-BPL synchronization algorithm for information exchange in herding operations
Source: PLoS One. 2025 Oct 22;20(10):e0334888. doi: 10.1371/journal.pone.0334888 (PMC12543189; doi:10.1371/journal.pone.0334888)
Supplement: Attachment — Submitted filename: Coomments-R1.pdf [file pone.0334888.s003.pdf]

Thank you for the revised manuscript and your responses. I appreciate the effort. However, many replies are brief and general, and they do not point to the exact places in the manuscript where changes were made. The paper still needs substantial refinement. Please treat the reviewers' suggestions as an opportunity to improve the work.

How to respond going forward: when you write "this has been addressed/clarified," please name the exact Section, Page, and Line numbers so the reviewer can verify the change quickly.

The following points refer to my earlier comments that have not yet been adequately addressed:

1. Comment #11 (notation $x_{ij}(t)$ vs $d_{ij}(t)$): In revised manuscript, Section 3.3.1 (p. 9, lines 12–13) you write that *$d_{ij}(t)$ is the binary decision variable for UAV movement (Euclidean distance)*. This is inconsistent. Is $d_{ij}(t)$ a distance (a real value) or a binary decision variable? If it is binary, how does it differ from $x_{ij}(t)$? Please clarify the definitions, use them consistently in text and equations, and ensure the paper reflects this correction.

2. Comment #14: I am not satisfied with the current response. Please revisit the original point and address it fully in the manuscript, indicating where changes were made (Section/Page/Line).

3. Comments #17 and #22 (references): I could not locate the sources you cited. Please provide precise citations (Section/Page/Line) and list the full references you used for the "quadcopter" claim.

4. Comment #10 ("critical data sources"): I could not find a clear answer. Please respond directly to this point in your rebuttal and update the manuscript accordingly, with exact locations of the changes.

5. Comment #20 (data path inconsistency): Your response says UAVs collect data from BPL relay points (aggregated sources), but the revised manuscript (Step 1 in your synchronization algorithm, p. 8) says UAVs collect directly from a sensor when priority $p_i(t) > \theta$. Please reconcile this: under what conditions do UAVs collect from relays vs directly from sensors? Align the algorithm and discussion so they tell one consistent story.

6. Comment #22 (runs vs iterations): You wrote "a multi-iteration simulation spanning 10 runs." Please clarify whether this means 10 runs (independent trials with results averaged) or 10 iterations within one run (time steps within a single simulation). Remember that they are different. Define run and iteration, what varies and what is fixed in your simulation, and reflect this clearly in the methods and results discussion.
