# Supplementary material for: Sky and wire: A UAV-BPL synchronization algorithm for information exchange in herding operations
Source: PLoS One. 2025 Oct 22;20(10):e0334888. doi: 10.1371/journal.pone.0334888 (PMC12543189; doi:10.1371/journal.pone.0334888)
Supplement: Attachment — Submitted filename: Response to Reviewers 2b.pdf [file pone.0334888.s004.pdf]

We would like to thank the Editorial team and anonymous reviewers for their feedback and comments, which helped improve this work. We have reviewed the reviewer's comments to clarify them and make necessary corrections. Our response to these comments is tabulated:

REBUTTAL FORM

REVIEWER A

S/N	COMMENT/OBSERVATION	RESPONSE
**1**	The current version is more refined as compared to the last time. The implementations scenarios have been addressed better in detail. However, there are typos in the current version that needs to be revised by the authors. Just a sample I am sharing<br><br>Consider a farm with N sensor nodes and M UAVs operating at 200*200m^2. what is this dimension? there seems to be some error in it. These kinds of technical typos need attention.	Thank you for the observation. The typo highlighted has been checked and corrected in section 3.3-line 2 (changes are highlighted). The entire manuscript has also been checked for typos and necessary corrections made.

REVIEWER B

S/N	COMMENT/OBSERVATION	RESPONSE
1	Comment #11 (notation $x_{ij}(t)$ vs $d_{ij}(t)$): In revised manuscript, Section 3.3.1 (p. 9, lines 12–13) you write that $d_{ij}(t)$ is the binary decision variable for UAV movement (Euclidean distance). This is inconsistent. Is $d_{ij}(t)$ a distance (a real value) or a binary decision variable? If it is binary, how does it differ from $x_{ij}(t)$? Please clarify the definitions, use them consistently in text and equations, and ensure the paper reflects this correction	Thank you for this comment. For clarity, this has been modified.<br>$d_{ij}(t)$ is the distance from UAV *j* to relay point that aggregates data from sensor and $x_{ij}(t)$ is the binary decision variable as presented in Section 3.3.1 (p. 9, lines 12–13) (recast)
2	Comment #14: I am not satisfied with the current response. Please revisit the original point and address it fully in the manuscript, indicating where changes were made (Section/Page/Line).	This was addressed fully in the revised manuscript under section 3, page 9, lines 1–12 of the revised manuscript.<br><br>Kindly note that the entire section was revised to capture your comment as well as other reviewers. To clarify further, the sensors generate the packets, which are considered and transmitted to the nearest relay point. The UAVs retrieve packets from the relay points.
3	Comments #17 and #22 (references): I could not locate the sources you cited. Please provide precise citations (Section/Page/Line) and list the full references you used for the "quadcopter" claim.	For comment 17, this was presented in the revised manuscript also under table 4, item 6 (simulation parameters). The reference to this was cited as [39] in the revised manuscript. Comment 22 was addressed in section 4 line 5 and satisfactory in its present form. Kindly refer

		to the relevant section of the manuscript highlighted in yellow.
4	Comment #10 ("critical data sources"): I could not find a clear answer. Please respond directly to this point in your rebuttal and update the manuscript accordingly, with exact locations of the changes	This was addressed in the revised manuscript. Section 3.1-line 4-7<br><br>The relay points consist of BPL modems equipped with isolation circuits and low-power WI-Fi modules.
5	Comment #20 (data path inconsistency): Your response says UAVs collect data from BPL relay points (aggregated sources), but the revised manuscript (Step 1 in your synchronization algorithm, p. 8) says UAVs collect directly from a sensor when priority $p_i(t) > \theta$. Please reconcile this: under what conditions do UAVs collect from relays vs directly from sensors? Align the algorithm and discussion so they tell one consistent story	Thank you for this comment. I understand the reviewer's concerns. I am kindly requesting the reviewer to read the statement again to understand that there are no inconsistencies. I specifically point the reviewer to the role of the priority here.<br>As stated in the revised manuscript, "If the packet has a high priority $p_i(t) > \theta$, UAVs are notified to collect data directly from the sensor.<br><br>Pertaining to your other observation "under what conditions do UAVs collect from relays vs directly from sensors?" kindly look at section 3.2 (UAV-BPL Synchronization) under item 1-5. This clearly explains this.
6	Comment #22 (runs vs iterations): You wrote "a multi-iteration simulation spanning 10 runs." Please clarify whether this means 10 runs (independent trials with results averaged) or 10 iterations within one run (time steps within a single simulation). Remember that they are different. Define run and iteration, what varies and what is fixed in your simulation, and reflect this clearly in the methods and results discussion.	It means 10 iterations within one run. The explanation provided in the result and discussion infers this based on the revised manuscript.<br><br>The word run has been revised to iterations for clarity<br>Section 4.1-line 5-page 12

REVIEWER C

S/N	COMMENT/OBSERVATION	RESPONSE
1	The authors introduce Sky and Wire, a synchronization algorithm that integrates Unmanned Aerial Vehicles with Broadband over Power Line (UAV-BPL) for herding operations. Two scenarios are analyzed: Scenario 1, the coverage-driven scenario and Scenario 2, the priority-driven exchange scenario. The algorithms corresponding to these scenarios are validated through MATLAB simulations.<br>Comments on the document:<br><br>Although the proposed UAV-BPL framework is very interesting and offers significant advantages, the document does not clearly explain how the proposed solution works. This lack of clarity is critical, since the main contributions of the paper are the algorithms themselves. A more detailed and consistent explanation is needed.	Thank you very much for this comment. The document has been re-written to ensure consistency. Modifications are highlighted in yellow.

2	For example, in Section 3.2, Phase 3, it is stated that the UAV transmits data collected from sensors to a nearby relay, which then forwards the data to cloud storage via BPL. However, just before, in Phase 2, the text states that data packets Pi are collected by the UAV either directly from sensors or FROM its designated RELAY, depending on the priority. It is therefore unclear why the UAV both collects data from relay points and transmits data to them.	Thank you for your observation. The section has been revised for consistency. Kindly refer to the following statement within the manuscript for clarity.<br><br>"As stated in the revised manuscript, "If the packet has a high priority $p_i(t) > \theta$, UAVs are notified to collect data directly from the sensor."
3	Figures 1–3 show no modifications; no improvements have been made to them (see previous comments).<br>In Equations (3) and (4), the symbols "x" and " . " appear, but their meaning is not explained. If both are simply multiplication signs, why the difference?<br>The definition of d_{ij} following Equation (3) has not been corrected.	Thank you. The quality of Figure 1-3 has been improved in terms of DPI. Figure 3 has been modified to capture the reviewer's suggestions. Same font text type has been used, and the word internet has been changed to cloud.<br><br>All parameters of the equations used have been defined in the text and are also presented in the Table 3, the table of the symbols.
4	The parameters used in all simulations should be clearly presented. What are the "random seeds" used for the presented results?	In all simulations we used the parameters already listed in Table 4 (farm size, number of sensors, UAV range, energy threshold, priority threshold, etc.). To account for stochastic effects such as random sensor placement and packet generation times, we ran each scenario for 10 iterations.
**5**	The presentation of results requires improvement (e.g., fonts, axis labels). In particular, the differences in UAV trajectories should be made more noticeable.	Thank you for this observation. All axes are now adequately labeled. Colored lines are now used. The black line shows the connection between the UAV and the relay node as a form of trajectory. This has been captured.
