# Supplementary material for: Sky and wire: A UAV-BPL synchronization algorithm for information exchange in herding operations
Source: PLoS One. 2025 Oct 22;20(10):e0334888. doi: 10.1371/journal.pone.0334888 (PMC12543189; doi:10.1371/journal.pone.0334888)
Supplement: Attachment — Submitted filename: Response to Reviewers.docx [file pone.0334888.s002.docx]

**REBUTTAL FORM**

**EDITOR**

| **S/N** | **COMMENT/OBSERVATION** | **RESPONSE** |
| --- | --- | --- |
| 1 | Please ensure that your manuscript meets PLOS ONE's style requirements, including those for file naming. | The manuscript has been formatted to meet the PLOS ONE’s style |
| 2 | If there are ethical or legal restrictions on sharing a de-identified data set, please explain them in detail | None |
| 3 | Please upload a copy of Figure 3, to which you refer in your text on pages 7, 12, and 13. | This has been uploaded |
| 4 | Please include a copy of Table 2, which you refer to in your text on pages 5 and 10 | This is included |

**REVIEWER 1**

| **S/N** | **COMMENT/OBSERVATION** | **RESPONSE** |
| --- | --- | --- |
| 1 | Improve the quality of figures | The Quality of the figures have been improved throughout the manuscript |
| 2 | What are the specific assumptions about the farm electrical infrastructure (e.g., BPL availability, grid reliability, noise/interference levels)? | The specific assumptions are included and presented in section 3.1 of the revised manuscript |
| 3 | How is the synchronization between UAV and BPL established and maintained in practice | This is explained in section 3.2 of the revised manuscript |
| 4 | Are there limitations on the number of UAVs or relay nodes? How scalable is the system for larger farms | There are no limitations to the number of UAVs nor relay nodes. however, as the number of UAVs increases, there was an increase in energy consumption and reduction in latency. This is discussed in section 4.2 of the revised manuscript. |
| 5 | What factors were considered when deciding the energy consumption model, and does it account for real UAV dynamics (e.g., wind, payload) | In response, we added in the Methodology section (Section 3) to clarify the factors considered in the energy model. The model assumes a constant energy consumption rate of 0.1% per 10 meters of UAV flight, based on typical quadcopter specifications (e.g., 500 Wh battery, 10 m/s speed) under controlled conditions. The reference is provided |
| 6 | How does the system prioritize data urgency ( ≥ 0.7)? What is the mechanism for determining urgency levels in real time? | This is captured in the 4^th^ line of the defined problem statement. |
| 7 | What happens if BPL communication is disrupted or the grid is unstable, are there fallback mechanisms? | In cases where BPL communication is disrupted or the grid is unstable, resulting in channel capacity falling below the minimum threshold UAVs temporarily store data onboard and retry transmission to a relay point when capacity is restored, as supported by IEEE 1901 standards for robust BPL recovery. This is captured under information exchange in section 3.2 of the revised manuscript |
| 8 | How does the proposed framework contribute to sustainable agriculture, especially in terms of reducing manual labor, improving animal welfare, or optimizing resource use | To address this, we have added a paragraph in the Conclusions section (Section 5) to explicitly outline its impact. The framework enhances sustainability by automating data collection from sensors across dairy farms, reducing manual labor |

**REVIEWER 2**

| **S/N** | **COMMENT/OBSERVATION** | **RESPONSE** |
| --- | --- | --- |
| 1 | Abstract is very poor and needs to convey the proposed idea or solution in a unique way | The Entire abstract has been re-written |
| 2 | Need to rewrite Introduction properly It’s a vague introduction Meaningless | Thank you. The introductory section has been revised |
| 3 | Conclusion needs to be revised and fine-tuned | The conclusions has been refined and finetuned |
| 4 | In addition, it has been observed that the authors do not demonstrate clearly and objectively the relevance of this research. When reading the research, we observed that the authors describe it subjectively, that is, the importance of the respective results, methodologies, and consequently of this article is not evident. Include recent references 2023/24 | The entire manuscript has been revised to capture this as well as the inclusion of recent references. |

**REVIEWER 3**

| **S/N** | **COMMENT/OBSERVATION** | **RESPONSE** |
| --- | --- | --- |
| 1 | The context and problem statement in the introduction are missing. It should be more elaborate | Thank you. The introductory section has been revised to address this |
| 2 | What is the focus and motivation of the work? These should be added in the introduction | This has been added in the introductory section |
| 3 | Improve by emphasizing the key novelties and contributions, incorporating essential numerical results. Ensure conciseness and focus. | To address this, we have revised the Introduction and Conclusions section (Section 5) to highlight the framework’s innovative integration of UAVs with BPL communication. |
| 4 | Clearly highlight research gaps, novelties, and contributions. | we have revised the Introduction, Problem statement and Conclusions section to highlight the framework’s innovative integration of UAVs with BPL communication. |
|  | Added the main contribution at the last of the introduction. | This was included earlier |
| 5 | The manuscript (Introduction) has deficient citations to very important  works published before; you can add these works:  <https://doi>.org/10.1016/j.iot.2025.101704;  <https://doi>.org/10.1016/j.sciaf.2024.e02527;  <https://doi>.org/10.1016/j.atech.2025.100979;  <https://ieeexplore>.ieee.org/document/10548972 ;  <https://link>.springer.com/chapter/10.1007/978-981-96-3094-3_10 | Some of the works have been checked in terms of its relevance to the research work and has been added (reference 23) |
| 6 | Rewrite the description of the pseudocode of the system in a  professional. | The pseudocode has been checked for potential errors and addressed professionally. |
| 7 | The Section of the Results and Discussion needs to add a more detailed description of the results presented in the figures and Tables should be provided, incorporating both qualitative and quantitative analysis. | The section of the result has been improved in the revised manuscript as suggested |
| 8 | Detail the approach used to validate numerical results. | The result was validated through 10 Monte Carlo simulation runs using MATLAB, with varying random seeds to ensure robustness |
| 9 | Please improve the conclusion section and add the Future Work. | The conclusion has been worked on and the recommendations for further work is presented in the conclusion section |

**REVIEWER 4**

| **S/N** | **COMMENT/OBSERVATION** | **RESPONSE** |
| --- | --- | --- |
| 1 | BPL integration with UAV is a promising concept to which I agree. Normally the transmission lines will be high power sources and directly connecting them with the IoT enabled fog devices for data extraction and forwarding could be dangerous. Is there a safe way to conduct this? I mean using step down transformers or some solution. Using UAVs to extract data and BPL points for forwarding and extracting must be studied from the perspective that what impact it will have on low power devices and at what level human interaction in this environment will take place as high voltage sources are involved. Therefore, the authors should address the safety and hazards along with this coupled scenario | Thank you. This has been captured in section 3.1 of the revied manuscript. |
| 2 | Table 2 has formatting issues please re format it. | Table 2 has been formatted as appropriate |
| 3 | In the coverage driven scenario 1 explain what equation 3 means clearly. The optimization equation must be linked and explained in light of equation 3. In the model what is the role for the redundancy. Is this something that you need meaning  that redundancy is required or this is some penalty value meaning that it is an overhead. This needs to be cleared. | The equations have been harmonized and reexplained in section 3.3.1 of the revised manuscript |
| 4 | Table is written again on the algorithm 1 of the coverage driven scenario. | This has been addressed |
| 5 | Line 22 of the algorithm 1 computes the average. What will you do with this and where is it used again in the algorithm? Is this value used in another algorithm? Please clearly mention it either before the algorithm or after it. | The pseudocode of the algorithm has been re-presented for clarity |
| 6 | The results of the study are good and are showing what the authors intends to achieve but the results should be clearly explained so that the reader is able extract what the lines means. For example, in figure 3 in scenario 1 and scenario 2 what is the meaning of black lines that are connected to the data sources and the BPL relays. What does this black means in both the scenarios there is no explanation related to that? | The results have been re-explained as suggested |

**REVIEWER 5**

| **S/N** | **COMMENT/OBSERVATION** | **RESPONSE** |
| --- | --- | --- |
| 1 | Based on the abstract, the research problem and research gaps have not been described. It is therefore difficult to discern what really motivated the development of the proposed framework. | The entire abstract has been re-written to address the observed concern |
| 2 | This paper used performance indicators such as coverage, energy usage, frequency of relay visits, and response timeliness using MATLAB simulations. What were the numerical values obtained for these indicators and what do these obtained values imply? | The explanation to this has been provided in section 4.2 of the revised manuscript |
| 3 | The main contributions of this paper reflect marginal novelty. You need to restructure this section so that the acclaimed novelty can be made clearer | This has been restructured |
| 4 | After Table 2, add a paragraph to describe a summary of the  identified research gaps. This should be followed by a clear explanation on how the proposed framework helps alleviate these gaps. | Thank you for the observation. This comment has been addressed after Table 2 |
| 5 | It is not clear why you are referring Algorithm 1 as Table 2.  Similarly, it is not clear why you are referring Algorithm 2 as Table 3. You already have Table 2 which gives a Summary of Relevant Works under Section 2. | This has been corrected |
| 6 | Ensure that all the algorithms are well discussed within text. | This has been addressed |
| 7 | Consider adding a table of all symbols and notations used throughout this paper. | This has been added and presented in Table 3 of the revised manuscript |
|  | In Table 4, you have given the Simulation Parameters. However, you have failed to describe the simulation tool/environment under which these parameters are deployed. | The simulation tools used was MATLAB this was stated in the manuscript |
|  | What informed the selection of the simulation parameters in Table 4? How will the results be affected if different values are deployed? | The simulation parameters were chosen intuitively and its effects are discussed in the result section |
|  | For each presented tables/graphs in section 4, given elaborate descriptions as well as interpretations. | This has been addressed |
|  | In the conclusion section, describe some of the limitations of the proposed framework. Thereafter, explain how these limitations may be addressed. | This has been discussed as advised in the conclusion section of the manuscript |

**REVIEWER 6**

| **S/N** | **COMMENT/OBSERVATION** | **RESPONSE** |
| --- | --- | --- |
| 1 | The abstract needs to be revisited. In the section where the two scenarios are described, the name of the first scenario differs from the one stated in the problem statement section. It's unclear where the description of scenario 2 begins, and the name of this scenario is not given in the abstract. | The Entire abstract has been revised |
| 2 | It is unclear what the advantage of using drones in this proposed algorithm is, and why the data from all sources is not sent directly to the farm operators or the cloud throughout BPL. The Introduction does not really set the work in context. | The introduction has been revised to address this concern |
| 3 | Several abbreviations without descriptions are used. There are some typos, for example, right before the table, of (of some?). | This has been corrected |
| 4 | The quality of the Figures should be improved, especially Fig. 3 (in this section, because Fig. 3 is repeated in the simulation and results section); there are blue boxes marked as “Repeater” instead of "relay points" as throughout the document. The fonts in the image have different sizes. In the document, "the cloud" is employed instead of "internet", as in the image. "LV line", "CPE", and "Head-End" are never defined. Suffixes "ij" on "d{ij}" are not defined, nor "Ø", "E_min", or "C_min". In equation (2), there is an extra ")" at the end. Right after this equation, E_i should be E_j. | The Quality of the figure is improved other corrections pointed out is addressed |
| 5 | In the proposed algorithm, the authors don't take into account the flight time consumption in the data transportation between relay points and UAVs. It should be mentioned why it is neglected. What are the communication protocols involved in the data collection?  The names of both scenarios differ throughout the document. Suffixes "ij" on "x{ij}" are not defined. In equations (3) and (8), the operator between x_{ij} and d_{ij} looks like a cross product, clarify. In the paragraph after equation (7), x_{ij} and d_{ij} have opposite definitions to the previous definitions. | Thank you. This is stated in section 3.2 of the revised manuscript.  Two-layer communication protocol.  Other observations have been addressed |
| 6 | Minimum energy is the total percentage or the used percentage? (Table 4). The initial conditions for UAVs are not given, nor is the starting point; if they are set randomly, this must be specified and justified. After Table 4, in the discussion of Fig. 3 of this section, relay spots and UAV initial positions are both described as red squares, which makes it impossible to understand. Then, it is said that the paths illustrate how the algorithms allocate UAVs to relays to maximize travel distance. This means that with less energy consumption, more distance can be covered? Explain in more detail. Fig. 3 is difficult to understand. For example, on Table 4, it is said that there are 10 BPL relay points; however, only 7 can be seen in each scenario, and the UAVs' initial positions are not shown. UAV paths are not dashed lines as described, and the path for each UAV can't be distinguished. The paragraph after Fig. 3 states that in scenario 2, 100% of high priority data is covered, whereas in the abstract, it's  stated that only 80% is covered.  The description of Fig. 4 states that Scenario 2 accounts for approximately 20% of total energy usage; however, it can be seen from Figure 4 that this is far from the 20% mark, closer to 10%. Maybe this kind of data could be better presented in a table with the exact value. Since it's a simulation, data should be accessed accurately. After Fig. 4, is it timelines or timeliness? Same on the title of Fig. 5.  typos: "secs" "moves". The 20 movements per UAV are not taken into account in the algorithms described above. Explain why this is required and how it is taken into account in the algorithms. Units on Fig. 5. Discussion about Fig. 6 states that the resource demand is highlighted; however, due to the scale, it's difficult to see the percentage of energy used, and it's not provided in the discussion. A Table with the actual value might be better. A deeper analysis is expected in the discussion of the results presented in Fig. 7 for Scenario 1. Why are there three relays with higher visitation frequency?  The bar graph of Scenario 1, Fig. 8, doesn't coincide with its description. The vertical scale in Fig. 8 is wrong. In general, as different data are presented for each scenario, it's difficult to compare them; similar data for both scenarios could say more about the main differences and advantages for a given need. | The initial conditions are chosen intuitively and are varied to see the effect.  The discussion section has been harmonized for clarity.  A table has been added to present the obtained data clearly.  The typos have been corrected.  A table has been included to address the stated concerns while a detail analysis is also presented for both scenario for better understanding. |
| 7 | The BPL dependability highlights the framework; however, the effect of the increased complexity of the wireless part of the framework is unclear. Limitations of the approach should also be mentioned. More simulations should be made, giving variations, for example, on the distributions of relay spots or the distribution of urgency, and the initial conditions of the drones, to arrive at the conclusions. | This has been addressed as presented in section 4.1 to 4.2 of the revised manuscript |

**REVIEWER 7**

| **S/N** | **COMMENT/OBSERVATION** | **RESPONSE** |
| --- | --- | --- |
| 1 | The abstract needs improvement in terms of language, flow, and clarity. Please revisit your abstract and rewrite it professionally and concisely, clearly highlight your objectives, contributions, and methodology. | The Entire abstract has been revised |
| 2 | You mentioned two scenarios in the abstract, but only one is  described, while the other is unclear and appears mixed into the description. | This has been addressed |
| 3 | The entire paper needs thorough English proofreading, as there are several unclear statements, improper flow, and ambiguities throughout the text. | The paper has been subjected to English proofreading |
| 4 | Apart from satellite communication, the paper requires more  statistical analysis regarding the cost-effectiveness and affordability of using UAVs compared to traditional wireless monitoring methods. | This has been done I believe in other papers which can be made reference to as this is not part of the scope of this work |
| 5 | Please correct “ ” in “Where ( ) is the energy level” to “ j” on page 8. | This has been corrected |
| 6 | Please remove the repetition in the fourth paragraph of Section 3.1, starting with “The aim is to minimize the total distance UAVs travel to…” | This has been corrected |
| 7 | Please revise the section on “The Coverage-Driven Scenario  (Scenario 1),” its equations, and the algorithm, as there are some contradictions between your descriptions, equations, and algorithm. For example, there is inconsistency regarding whether UAVs collect data from sensors or relay points. | This has been revised |
| 8 | It is not clear how BPLs are used, what standards are followed, and how they operate. Please elaborate and provide technical details on BPL operation and its integration with UAVs. | This has been provided in the revised manuscript under section 3.1 and 3.2 |
| 9 | There should be a separate section explaining the “UAV-BPL synchronization” in detail, including how it is achieved and what are the transmission protocols used from sensors to BPLs, through BPLs to its relay points, and from relay points to UAVs. | This section has been included as section 3.1 in the revised manuscript |
| 10 | What are relay points made of? Please clarify. | This has been clarifies and stated in the revised manuscript |
| 11 | There seems to be a contradiction between your objective equation no. 3 and the description of xij and dij . Based on equation 4, I assume “xij(t) is the binary decision variable for UAV movement (xij(t) =1 if UAV j collects data from source i, 0 otherwise),” and “dij(t) is the distance from UAV j to the BPL relay serving source i” correct? This is different from your description. Please revisit all your equations and descriptions and clarify the relationship between variables for the reader. | All equations have been revisited and revised |
| 12 | Also, in Algorithm 1, there are contradictions: “( )” is used as the distance to the nearest relay, while xij(t) is used as a decision variable. Please ensure consistency. | This has been revised for consistency |
| 13 | I did not see a check for CBPL(t)≥Cmin before relaying data, as indicated in line 17 of the algorithm 1. | This has been revised in the pseudocode |
| 14 | It is unclear what is considered a data source. In Section 3, you mention data sources 1 to N (sensors) creating packets and sending them to relay points, but later in the scenario description, it seems you refer to relay points as data sources visited by UAVs. Please be consistent. | This has been revised for consistency |
| 15 | I did not see any UAV energy/range check in Algorithm 1. Is it used as input? Why? | This has been revised in the pseudocode in Algorithm 1 |
| 16 | I suggest using a threshold symbol other than ∅, which means “empty set” | Thank you. The suggestion has been addressed |
| 17 | In Algorithm 2, the energy consumption rate is used, but how is it determined? Please explain or provide a proper citation. | In response, we added in the Methodology section (Section 3) to clarify the factors considered in the energy model. The model assumes a constant energy consumption rate of 0.1% per 10 meters of UAV flight, based on typical quadcopter specifications. The reference is provided |
| 18 | Please remove the two captions “Table 2 Coverage-Driven” and “Table 3 Energy-Efficient Priority-Driven Exchange.” You can refer to them as Algorithm 1 and 2, which are already captioned. | Thank you. The suggestion has been implemented in the revised copy |
| 19 | How do you define "critical" data sources, or in what cases we know that it is critical? and how is R chosen? | This as been addressed and discussed in section 3 of the manuscript |
| 20 | Does your model allow a UAVs to visit more than one data source per time step? If so, should there be a constraint on UAVs capacity or energy? | we have added a paragraph in the Methodology section (Section 3.2, UAV Path Planning) to clarify the model’s approach. In the model, UAVs collect data from BPL relay points aggregating multiple sensor data sources, not directly from individual sensors per time step, as sensors are assigned to the nearest relay based on Euclidean distance |
| 21 | In your simulation, you mention a 200x200 area. Is this in meters? If so, this is a small-scale farm that could be covered by traditional wireless methods, while your solution aims to address large-scale farms. How do you validate that your solution meets your design objectives? | Yes, it is in meters.  To clarify, the 200×200 m² area used in the simulations is indeed in meters, representing a medium-sized dairy farm for baseline testing, as noted in the Methodology section (Section 3). To address scalability for larger farms, we have added a paragraph in the Simulation Results and Discussion section (Section 4.3, Scalability and System Limitations) to validate the framework’s performance. Simulations were extended to larger farm sizes (up to 400×400 m²), showing that the framework maintains adequate coverage and a response time for high-priority data, with latency increasing and energy consumption. |
| 22 | This simulation is conducted for a single iteration of target monitoring, which definitely results in higher performance metrics and very low energy consumption. For validity, the simulation should be extended to cover a specified duration that emulates several hours or days of operation. | we have revised the Simulation Results and Discussion section (Section 4.1) to include a multi-iteration simulation spanning 10 runs to ensure robustness |
| 23 | Your simulation lacks context and a valid environment. Please mention your simulation environment (tools, libraries), and explain how you simulate UAVs and BPLs, the standards and protocols assumed for communication and transmission, and the integration of UAVs and BPLs. Even though you have “Communication Technologies Comparison” in you related work section, but these were absent in you simulation part. | To address this, we have added a paragraph in the Simulation Results and Discussion section (Section 4) to detail the simulation setup. Simulations were conducted in MATLAB R2022b, using built-in functions and rng for reproducibility, without additional external libraries. UAVs are simulated as mobile agents with random initial positions, Euclidean distance-based path planning to relays (within 100 m range), and energy depletion at 0.1% per 10 m flight. |
| 24 | The simulation also lacks in-depth analysis, comparison, and justification. It appears that results are based on set parameters without reference points. Please provide more analysis and justification. | we have expanded the Simulation Results and Discussion section with a detailed analysis of key metrics, comparisons to reference points from existing methods, and justifications for parameter choices. |
| 25 | In your simulation results, you claim “100% coverage of all 50 data sources” and “highlighting its energy efficiency”. This result is expected given your design, implementation, and parameter selection, but there is no comparison with other solutions to demonstrate novelty. Please provide comparisons with some existing approaches and highlight where your solution is superior. | To address this, we have expanded the Simulation Results and Discussion section. |
| 26 | What energy consumption model did you use? | In response, we added in the Methodology section (Section 3) to clarify the factors considered in the energy model. The model assumes a constant energy consumption rate of 0.1% per 10 meters of UAV flight, based on typical quadcopter specifications (e.g., 500 Wh battery, 10 m/s speed) under controlled conditions. The reference is provided |
| 27 | How did you choose the parameters in Table 4 (UAV range, energy rate, redundancy factor, critical data, etc.)? Are they based on datasheets, chosen arbitrarily, or taken from references? | The simulation parameters are chosen intuitively and varied for performance |
| 28 | In Figure 1, the legend does not reflect what is shown in the figure. For example, all UAVs 1 to 5 have the same line style and color, so why you listed them separately? Also, the colors (green, blue-green, yellow, etc.) given on the scale 0.2 to 0.8 are not explained. | This has been addressed |
| 29 | I also suggest diversifying your results presentation by including tables along with graphs. | This has been addressed as suggested |
| 30 | Please use a standard referencing style according to the journal guidelines. | The referencing section has been formatted according to guidelines (Vancouver) |
